# Alien and native species in Italian marine and transitional waters

**DOI:** 10.3897/BDJ.11.e101464

**Published:** 2023-04-27

**Authors:** Cristina Di Muri, Tamara Lazic, Ilaria Rosati, Cataldo Pierri, Angela Boggero, Giuseppe Corriero, Alberto Basset

**Affiliations:** 1 National Research Council (CNR), Research Institute on Terrestrial Ecosystems (IRET), Lecce, Italy National Research Council (CNR), Research Institute on Terrestrial Ecosystems (IRET) Lecce Italy; 2 LifeWatch ERIC, Lecce, Italy LifeWatch ERIC Lecce Italy; 3 University of Bari, Bari, Italy University of Bari Bari Italy; 4 LifeWatch Italy, Lecce, Italy LifeWatch Italy Lecce Italy; 5 National Research Council (CNR), Water Research Institute (IRSA), Verbania, Italy National Research Council (CNR), Water Research Institute (IRSA) Verbania Italy; 6 University of Salento, Lecce, Italy University of Salento Lecce Italy

**Keywords:** non-indigenous species, introduced species, biodiversity, transitional ecosystems, marine ecosystems, EUNIS habitats, LifeWatch

## Abstract

**Background:**

Biological invasions are one of the major threats to the ecosystem structure and functioning. After the initial introduction, frequently mediated by human activities, alien species can overcome different biogeographical and ecological barriers and determine severe impacts on native biodiversity and socio-economic activities. The Italian peninsula is located at the intersection of large trade routes within the Mediterranean Sea. Such position, along with the intense commercial activity and the high population density of the Italian coast, are considered important drivers of alien species in Italian marine and transitional ecosystems. The Italian peninsula, however, is also one of the regions with the highest native species richness within the Mediterranean Sea and, therefore, it is crucial to account for both alien and native species diversity when estimating the impact of biological invasion. Yet, such comprehensive information is frequently scattered across several biodiversity information systems and databases.

**New information:**

Here, two datasets with alien and native species records in Italian marine and transitional waters are described. These datasets, created for the LifeWatch Italy case study on alien species, are the result of a large-scale collaboration involving experts working across the whole range of taxonomic diversity. The marine dataset includes a total of 12,219 records belonging to 3,772 species gathered from 91 investigated sites and seven EUNIS habitats. The dataset on transitional waters biodiversity includes 3,838 records belonging to 2,019 species found in 23 locations and four EUNIS habitats. Alien species were recorded in both marine and transitional waters, accounting respectively for 140 and 171 biological records belonging to 59 and 97 species. These occurrence data can be used for further research studies or management purposes, including the evaluation of the invasion risk and the formulation of alien species control and management plans. Furthermore, these compiled datasets can be used as input data for the Biotope vulnerability case study of LifeWatch ERIC, which offers a number of ICT services for the calculation of the incidence and of the impact of alien species on European biotopes.

## Introduction

Biological invasions are a major driver of ecosystem changes (Pyšek et al. 2020) and the dispersal of alien and invasive species in aquatic ecosystems have already determined severe impacts upon ecosystems functions and biodiversity as well as on socio-economic activities (e.g. Katsanevakis et al. (2014), Morri et al. (2019)).

The Mediterranean Sea is considered a hotspot of biological invasions (Bailey et al. 2020, Galil et al. 2018). Throughout time, the Mediterranean has experienced drastic biological changes, occasionally consisting in the replacement of most of its biota. These changes, however, have occurred naturally over thousands of years due, for example, to extreme variations in environmental conditions (Rilov and Galil 2009). In the anthropocene, human-mediated activities are determining comparable biodiversity changes that are taking place at a much faster pace and over shorter timescales (Giangrande et al. 2020, Rilov and Galil 2009). The opening of the Suez Canal in 1869 has facilitated the arrival of new species and climate change is fostering further aided and unaided migrations in the Mediterraneanean which, over the last five decades, has experienced the highest rate of biological introductions (Galil et al. 2018, Mannino et al. 2017, Zenetos et al. 2017).

In the Mediterranean Sea, coastal areas and, particularly, transitional water ecosystems such as estuaries, lagoons and coastal lakes, are largely exploited for their resources as well as for fisheries and acquaculture activities (Pérez-Ruzafa and Marcos 2012). The anthropogenic pressure on such ecosystems has resulted in the degradation and loss of habitats, as well as in the extirpation of local biodiversity, which has been further threatened by the large number of bioinvasions (Lotze et al. 2006). Despite transitional water ecosystems covering only a small fraction of the Mediterranean's coastline, they provide more ecosystem services and societal benefits than other aquatic ecosystems and hold numerous endemisms and unique species assemblages (Chapman 2012, Elliott and Whitfield 2011). Transitional waters are, however, particularly susceptible to biological invasions as they offer natural refugees and favourable conditions for the arrival and settlement of new species (Occhipinti-Ambrogi et al. 2010).

The Italian peninsula, with its 171 transitional water bodies spread along 7,000 km of coastline (Cardone et al. 2013, Servello et al. 2019), lies in a strategic position within the Mediterranean Basin as it is located at the crossroads of three different subregions (Directive 2008/56/EC, European Parliament (2008)) and at the intersection of water masses with different hydrographic conditions (Corriero et al. 2015, Occhipinti-Ambrogi et al. 2010). Italy represents the main passageway of the maritime traffic within the Mediterranean and such pathway is responsible for half of the total alien species introductions along the Italian coast (Servello et al. 2019). Notwithstanding their high invasibility, Italian coastal and transitional waters also harbour a rich native biological diversity (Bianchi and Morri 2000). Native species diversity and community composition are considered key factors in the ecosystem responses to biological invasions; hence, it is crucial to take these ecosystem components into account when studying the impact of biological invasions within a certain geographic area or habitat (Corriero et al. 2015, Colangelo et al. 2017). To date, a number of studies focused on the collection and publication of alien and invasive species checklists along the Italian coast (Occhipinti-Ambrogi et al. 2010, Servello et al. 2019). However, there is a lack of geo-referenced datasets reporting comprehensive biodiversity information including records of native and alien species across a broad range of taxonomic groups and locations in Italian marine and transitional waters.

The access to accurate and comprehensive species distribution data is crucial to evaluate spatio-temporal changes in biodiversity patterns, as well as the impact of alien species. Detailed information of species occurrence can improve our ability to quickly react and mitigate the challenge of biological invasions. Biodiversity data, however, are usually spread over a number of different platforms (e.g. GBIF, EMODnet, OBIS) and it is often difficult for scientists and stakeholders to access such an enormous amount of heterogeneous and diverse information. Furthermore, several international open-access databases, such as the World Register of Marine Species, FishBase and AlgaeBase, gather biodiversity information focusing on a single taxonomic group or ecosystem and it can be difficult to simultaneously extract and combine data from different sources into unique and standardised datasets for different taxa and locations of interest. Here, two datasets with geo-referenced occurrence records in Italian marine and transitional waters are described. The presented datasets include comprehensive biodiversity information belonging to different taxonomic groups (i.e. phytoplankton, algae, invertebrates and fish) and cover several habitats and regions all along the Italian peninsula. In both datasets, each record is associated with the status information, i.e. if the taxon is considered alien or native in the geographic area in which it occurs. Moreover, for each geo-referenced occurrence, the EUNIS habitat type is defined.

## General description

### Purpose

In this paper, two datasets with occurrence records of native and alien species in Italian marine and transitional ecosystems are presented. These datasets are two research products generated within the LifeWatch Italy case study on alien species. The coordination team of this case study, with the collaboration of a larger interest group, developed between 2013 and 2015, a series of resources and facilities for the research community engaged with alien species and available through the LifeWatch Italy website. The research facilities include a virtual research environment, a thesaurus and five biodiversity datasets with native and alien species occurrences. Altogether these resources contribute to an enhanced access and (re)use of scientific information on alien and native biodiversity on the Italian peninsula. Here, the marine and the transitional waters biodiversity datasets are described in detail. The marine dataset includes more than 12,200 records distributed over 91 sites and seven EUNIS habitats (level 2) and the transitional waters dataset holds over 3,800 records gathered from 23 sites and four EUNIS habitats (levels 2 and 3). These occurrence collections provide accurate and verified distribution information that could be used by the scientific community in further research studies and by stakeholders and policy-makers to define conservation priorities, to improve monitoring programmes and to enforce the current regulation with the aim of mitigating the risk of new invasions and of controlling the spread of established alien species. Additionally, these datasets can be used as input files for the Biotope vulnerability workflow developed within the Internal Joint Initiative of LifeWatch ERIC. Such analytical workflow uses native and alien species occurrences to estimate the incidence and the impact of alien species on different European habitats.

## Project description

### Study area description

**Figure 1.**
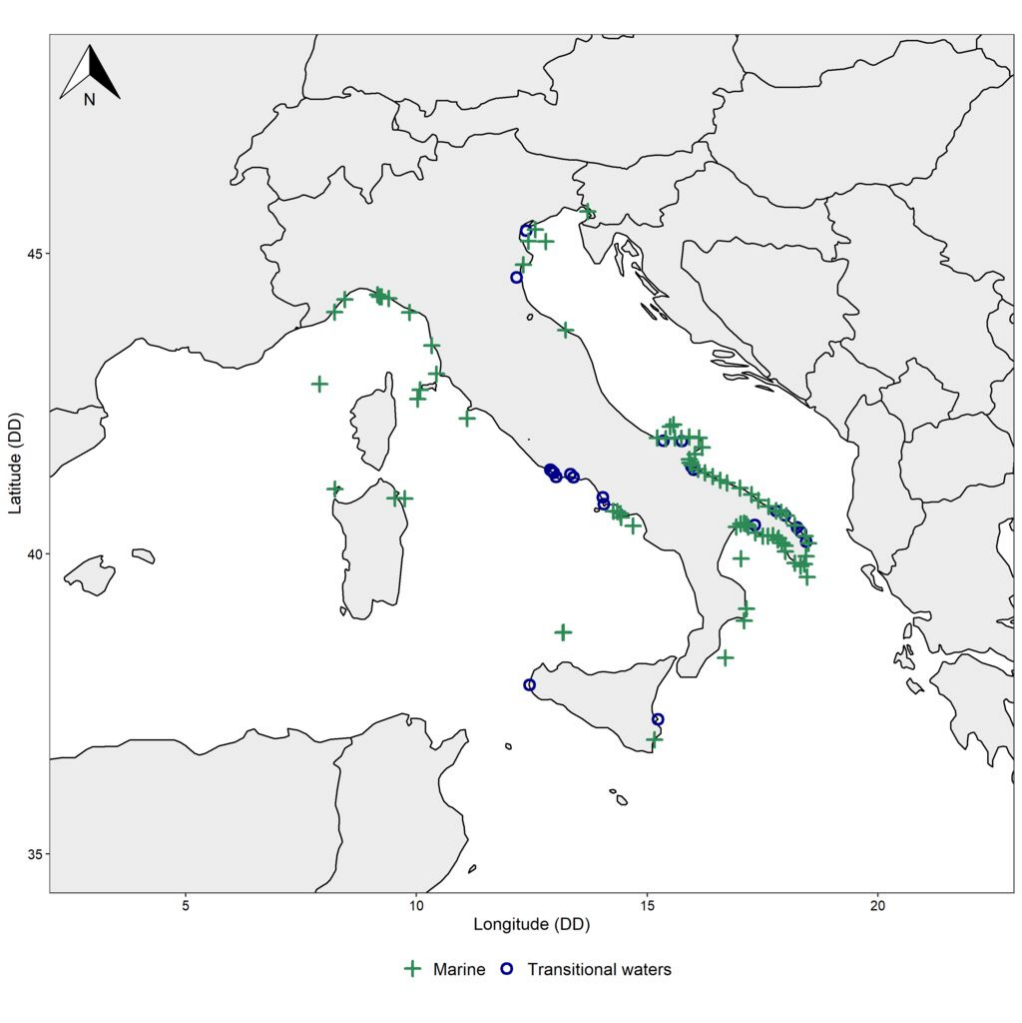
Map of the marine (green crosses) and transitional (blue circles) locations included in the datasets.

Marine and transitional areas of the Italian peninsula (Fig. 1). The occurrence records included in the datasets are widely distributed along the coast of Italy, encompassing the Ligurian, Tyrrhenian, Ionian and Adriatic Seas and spanning from Friuli Venezia Giulia to Sicily (northernmost to southernmost locations) and from Sardinia to Apulia (westernmost to easternmost locations). However, some geographical areas are more covered than others (Fig. 1) as occurs in most national and international biodiversity datasets. The uneven distribution of biodiversity records is inevitably associated with a better biological and ecological understanding of some biogeographical areas at both national and global levels. Our comprehensive collection of biological information can, therefore, also be used to inform environmental managers and stakeholders that more intense biomonitoring efforts should be directed towards those areas that are currently understudied.

### Design description

Occurrence records were gathered from different research institutions and consortia associated with LifeWatch Italy (Table 1) and have been used in published studies (check, for example, Boggero et al. (2014), Corriero et al. (2015)) to assess the habitat vulnerability to alien species within the framework of the LifeWatch Italy Alien Species use case. In both datasets, each record is associated with taxonomic information (i.e. species, genus, family, order, class, phylum, kingdom) and sampling event details including date, location and geographical coordinates. Furthermore, the EUNIS classification system was used to define the type of habitat and species group associated with each record and an additional column was used to specify whether the species is considered alien in the corresponding location of the Italian coast. The adopted definition of alien species is available in the Alien Species Thesaurus produced by LifeWatch Italy, according to which, a species is considered alien if it is deliberately or inadvertently introduced by human activities outside its past or current distribution (Boggero et al. 2014).

## Sampling methods

### Study extent

The occurrence records included in the marine dataset were collected from 1985 to 2015, whereas the records included in the dataset on transitional ecosystems are from 1940 to 2015.

### Sampling description

Taxonomic records were gathered from datasets belonging to several public and private research institutions (Table 1).

### Quality control

After data collection, all records were standardised and validated. Different platforms were queried to check for taxonomic reliability and taxonomic consistency, including the taxa-match tools available in the Pan-European Species directories Infrastructure (PESI), World Register of Marine Species (WoRMS) and Catalogue of Life (CoL). Specifically, PESI was initially used for the taxonomic matches, when a match was not found, WoRMS was queried and, in case WoRMS was not responsive, CoL was used at last. Datasets’ curation was then followed by a final check carried out by Italian taxonomic experts of each specific taxonomic group (i.e. phytoplankton, fish, algae, invertebrates). The taxonomic experts contributed to the validation of each record by: 1. evaluating if the reported occurrence for geographic area was reliable and 2. assigning the status of the taxon (alien vs. native) in that area.

## Geographic coverage

### Description

**Figure 2.**
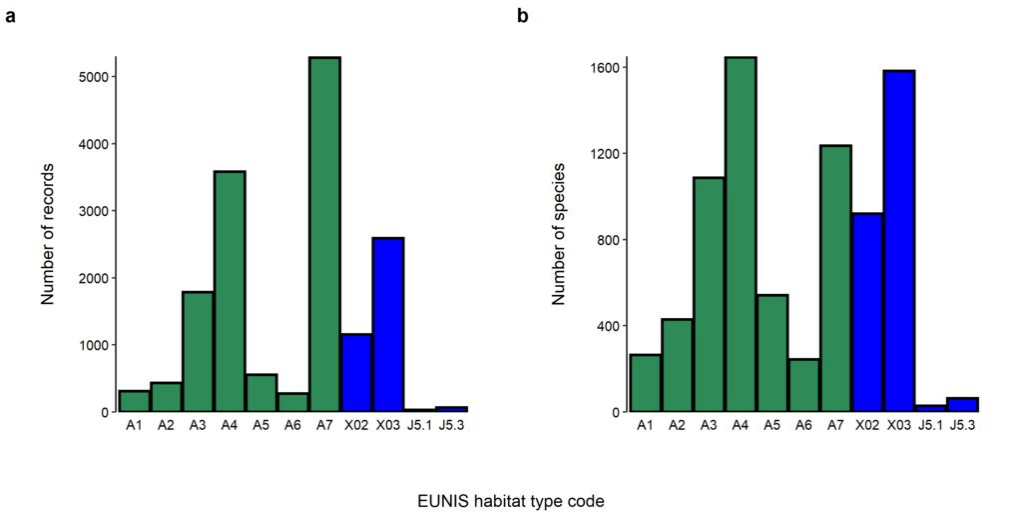
Barplots showing the number of records (a) and the number of species (b) per EUNIS habitat type included in the marine (green bars) and transitional waters (blue bars) datasets.

The marine dataset includes biological records gathered from 91 sites (Bounding coordinates 45.70080 N, 36.900000 S, 18.493927 E, 7.89840 W), whereas biological records of the transitional waters dataset were collected across 23 sites (Bounding coordinates 46.9818 N, 36.900000 S, 18.493927 E, 7.3920000 W). All locations within the datasets were classified according to the European Nature Information System (EUNIS) and considering the second and third level of the classification structure (Table 2). According to such classification system, the highest number of records for the marine dataset was found in the pelagic water column (EUNIS habitat code A7; Fig. 2a), followed by the circa-littoral rock and other hard substrata (EUNIS habitat code A4; Fig. 2a) and the infra-littoral rock and other hard substrata (EUNIS habitat code A3; Fig. 2a). The highest number of marine species was recorded in the circa-littoral rock and other hard substrata (EUNIS habitat code A4; Fig. 2b), followed by the pelagic water column (EUNIS habitat code A7; Fig. 2b) and the infra-littoral rock and other hard substrata (EUNIS habitat code A3; Fig. 2b). As for the transitional waters dataset, the majority of biological records and species occurred in coastal brackish habitats (EUNIS habitat code X03; Fig. 2) as opposed to saline coastal lagoons (EUNIS habitat code X02; Fig. 2).

### Coordinates

36.519702 and 45.981695 Latitude; 6.679688 and 18.632813 Longitude.

## Taxonomic coverage

### Description

**Figure 3.**
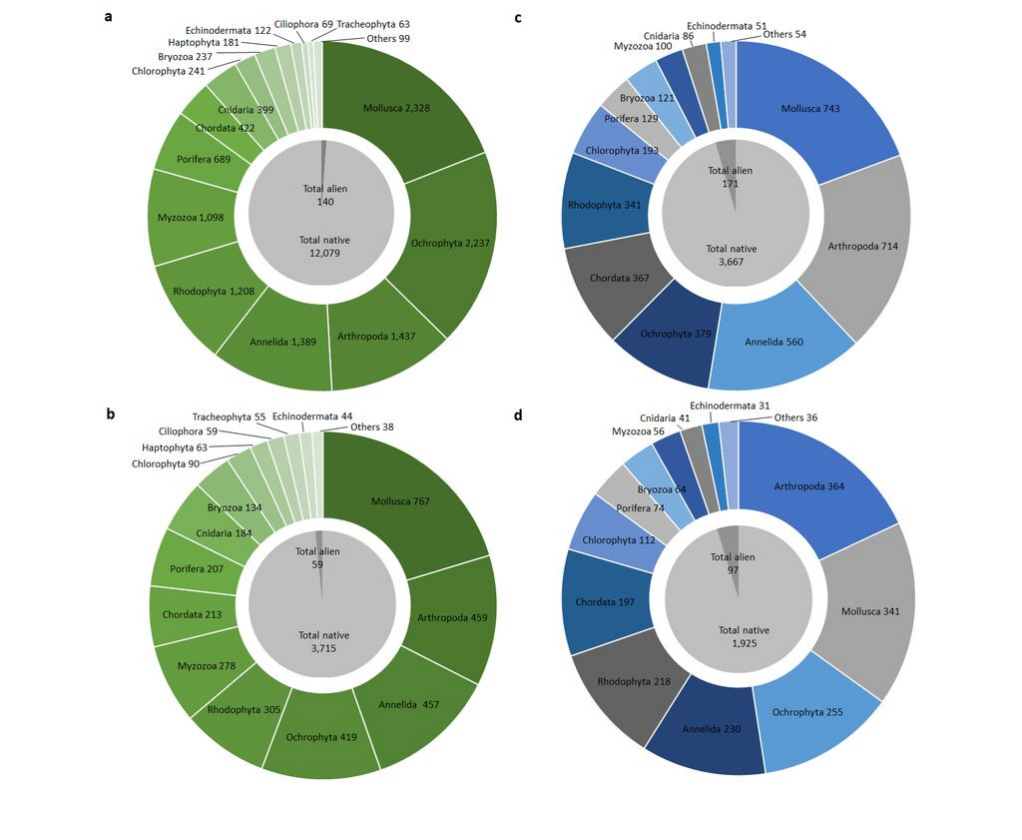
Donut charts showing the number of records (a, c) and the number of species (b, d) per phylum included in the marine (green; a, b) and transitional waters (blue; c, d) datasets. The pie charts within the donut charts represent the total number of alien and native records/species for each dataset.

The marine dataset contains 12,219 occurrence records belonging to 3,772 species (3,715 native and 59 alien species) and 16 phyla (Fig. 3a, b). The highest number of both records and species was recorded in the phylum Mollusca, followed by Ochrophyta, Arthropoda, Annelida and Rhodophyta (Fig. 3a, b). The transitional waters dataset contains 3,838 occurrence records belonging to 2,019 species (1,925 native and 97 alien species) and 13 phyla (Fig. 3c, d). The highest number of records was found in the phylum Mollusca, followed by Arthropods, Annelida, Ochrophyta and Chordata, while the highest number of species was recorded in the phylum Arthropoda, followed by Mollusca, Ochrophyta, Annelida and Rhodophyta (Fig. 3c, d). It should be noted that the difference in numbers between total species across all phyla and total alien and native species is due to the status of some species which are considered native in some localities and alien in others. Specifically, such species are *Ebriatripartita* and *Monocorophiumsextonae* for the marine dataset and *Acanthophoranayadiformis*, *Amphibalanuseburneus* and *Erythrocladiairregularis* for the transitional waters dataset.

## Usage licence

### Usage licence

Creative Commons Public Domain Waiver (CC-Zero)

### IP rights notes

This work is licensed under the Creative Commons Attribution 4.0 International Licence. To view a copy of this licence, visit http://creativecommons.org/licenses/by/4.0/.

## Data resources

### Data package title

Biodiversity data of Italian marine and transitional ecosystems gathered for the alien species study case of LifeWatch Italy.

### Alternative identifiers

Marine dataset: https://doi.org/10.48372/30d8844e-1774-4c63-89c4-4d858f88317d; Transitional waters dataset: https://doi.org/10.48372/98914415-765c-4650-b272-b16793231e8a

### Number of data sets

2

### Data set 1.

#### Data set name

Biodiversity data of Italian marine ecosystems gathered for the alien species study case of LifeWatch Italy.

#### Data format

CSV

#### Download URL

https://dataportal.lifewatchitaly.eu/view/urn%3Auuid%3A9f57ac94-8755-4991-b1b4-fe4e8717ec3d

#### Description

The dataset contains over 12,200 alien and native species occurrence records distributed across 91 marine sites and seven EUNIS habitats (level 2) along the Italian coast.

**Data set 1.** 

Column label	Column description
catalognumber	An identifier (preferably unique) for the record within the data set or collection.
eventdate	The date-time or interval during which an Event occurred. For occurrences, this is the date-time when the event was recorded.
waterbody	The name of the water body in which the Location occurs.
locality	The specific description of the place. This term may contain information modified from the original to correct perceived errors or standardize the description.
decimallatitude	The geographic latitude of the geographic center of a Location (in decimal degrees, using the spatial reference system WGS84). Positive values are north of the Equator, negative values are south of it. Legal values lie between -90 and 90, inclusive.
decimallongitude	The geographic longitude of the geographic center of a Location (in decimal degrees, using the spatial reference system WGS84). Positive values are north of the Equator, negative values are south of it. Legal values lie between -90 and 90, inclusive.
phylum	The full scientific name of the phylum or division in which the taxon is classified.
class	The full scientific name of the class in which the taxon is classified.
order	The full scientific name of the order in which the taxon is classified.
family	The full scientific name of the family in which the taxon is classified.
genus	The full scientific name of the genus in which the taxon is classified.
providedscientificname	The scientific name, with authorship and date information if known, as it originally appeared before of the taxonomic check.
scientificname	The full scientific name, with authorship and date information if known. When forming part of an Identification, this should be the name in lowest level taxonomic rank that can be determined. This term should not contain identification qualifications, which should instead be supplied in the IdentificationQualifier term.
scientificnameauthorship	The authorship information for the scientificName formatted according to the conventions of the applicable nomenclaturalCode.
eunishabitatstypecode	Assignment of the habitat type code based on the EUNIS habitat classification.
alien	A species, subspecies or lower taxon, introduced outside its natural past or present distribution; includes any part, gametes, seeds, eggs, or propagules of such species that might survive and subsequently reproduce.
eunisspeciesgroups	Assignment of the organism group based on the EUNIS species groups.
namepublishedinyear	The four-digit year in which the scientificName was published.

### Data set 2.

#### Data set name

Biodiversity data of Italian transitional ecosystems gathered for the alien species study case of LifeWatch Italy.

#### Data format

CSV

#### Download URL

https://dataportal.lifewatchitaly.eu/view/urn%3Auuid%3Ab5e33b56-752c-4fe7-9a53-face2497e40c

#### Description

The dataset contains over 3,800 alien and native species occurrence records distributed across 23 transitional waters sites and four EUNIS habitats (levels 2 and 3) along the Italian coast.

**Data set 2.** 

Column label	Column description
catalognumber	An identifier (preferably unique) for the record within the data set or collection.
eventdate	The date-time or interval during which an Event occurred. For occurrences, this is the date-time when the event was recorded.
locality	The specific description of the place. This term may contain information modified from the original to correct perceived errors or standardize the description.
decimallatitude	The geographic latitude of the geographic center of a Location (in decimal degrees, using the spatial reference system WGS84). Positive values are north of the Equator, negative values are south of it. Legal values lie between -90 and 90, inclusive.
decimallongitude	The geographic longitude of the geographic center of a Location (in decimal degrees, using the spatial reference system WGS84). Positive values are north of the Equator, negative values are south of it. Legal values lie between -90 and 90, inclusive.
kingdom	The full scientific name of the kingdom in which the taxon is classified.
phylum	The full scientific name of the phylum or division in which the taxon is classified.
class	The full scientific name of the class in which the taxon is classified.
order	The full scientific name of the order in which the taxon is classified.
family	The full scientific name of the family in which the taxon is classified.
genus	The full scientific name of the genus in which the taxon is classified.
providedscientificname	The scientific name, with authorship and date information if known, as it originally appeared before of the taxonomic check.
scientificname	The full scientific name, with authorship and date information if known. When forming part of an Identification, this should be the name in lowest level taxonomic rank that can be determined. This term should not contain identification qualifications, which should instead be supplied in the IdentificationQualifier term.
scientificnameauthorship	The authorship information for the scientificName formatted according to the conventions of the applicable nomenclaturalCode.
eunishabitatstypecode	Assignment of the habitat type code based on the EUNIS habitat classification.
alien	A species, subspecies or lower taxon, introduced outside its natural past or present distribution; includes any part, gametes, seeds, eggs, or propagules of such species that might survive and subsequently reproduce.
eunisspeciesgroups	Assignment of the organism group based on the EUNIS species groups.
namepublishedinyear	The four-digit year in which the scientificName was published.

## Figures and Tables

**Table 1.** List of LifeWatch Italy institutions involved in the biodiversity data collections for the two published datasets.

**LifeWatch Italy institutions**
Apulian Regional Agency for the Environmental Prevention and Protection (ARPA)
Centre for Estuarine and coastal MArine Sciences (CEMAS) of Venice
National Institute of Oceanography and Experimental Geophysics (OGS) of Trieste
National Research Council (CNR), Institute for Coastal Marine Environment (IAMC) of Taranto and Castellammare del Golfo now Water Research Institute (IRSA)
National Research Council (CNR), Institute of Marine Science (ISMAR) of Venice
National Research Council (CNR), Water Research Institute (IRSA) of Verbania
Polytechnic University of Marche
Sapienza University of Rome
University of Bari "Aldo Moro"
University of Ferrara
University of Genoa
University of Perugia
University of Salento
University of Sassari
Zoological Station "Anton Dohrn" of Naples

**Table 2.** List of EUNIS habitats included in the datasets with biological records in Italian marine and transitional waters.

EUNIS CODE	LEVEL	HABITAT TYPE NAME	DATASET
A1	2	Littoral rock and other hard substrata	Marine
A2	2	Littoral sediment	Marine
A3	2	Infra-littoral rock and other hard substrata	Marine
A4	2	Circa-littoral rock and other hard substrata	Marine
A5	2	Sublittoral sediment	Marine
A6	2	Deep-sea bed	Marine
A7	2	Pelagic water column	Marine
X02	2	Saline coastal lagoons	Transitional
X03	2	Brackish coastal lagoons	Transitional
J5.1	3	Highly artificial saline and brackish standing waters	Transitional
J5.3	3	Highly artificial non-saline standing waters	Transitional
